# Enrichment of Retroviral Sequences in Brain Tissue from Patients with Severe Demyelinating Diseases

**DOI:** 10.16966/2473-1846.132

**Published:** 2017-07-16

**Authors:** JD Kriesel, PJ Bhetariya, BK Chan, T Wilson, KF Fischer

**Affiliations:** 1 Department of Internal Medicine, Division of Infectious Diseases, USA; 2 Yale University, Department of Ecology and Evolutionary Biology, New Haven, Connecticut, USA; 3 Department of Pathology, University of Utah School of Medicine, Salt Lake City, Utah, USA

**Keywords:** Deep sequencing, RNA-seq, Next generation sequencing, Endogenous retroviruses, HERV, Multiple sclerosis, Progressive MS

## Abstract

**Background:**

Our group has used deep sequencing to identify viral RNA signatures in human brain specimens. We have previously used this method to detect HSV1, GBV-C, and measles virus sequence in brain tissue from deceased donors. Deep sequencing was performed on brain specimens from a cohort of patients who died with progressive forms of MS, revealing evidence of increased expression of some human endogenous retrovirus (HERV) domains.

**Objectives:**

Identify RNA sequences and new antigens involved in the pathogenesis of MS

**Methods:**

Deep sequencing was performed on RNA extracted from 12 progressive MS, 2 neuromyelitis optica (MS/NMO = demyelination group), 14 normal control, and 7 other neurologic disease (OND) control frozen brain specimens. The resulting single-ended 50 bp sequences (reads) were compared to a non redundant viral database representing (NRVDB) all 1.2 M viral records in GenBank. A retroviral gene catalog (RVGC) was prepared by identifying human genetic loci (GRCh37.p13) homologous to domains contained in the Gypsy 2.0 retro element database. Reads were aligned to the RVGC and human transcriptome with Bowtie2. The resulting viral hit rates (VHRs) were normalized by the number of high quality reads. The expression of human genes, including HERVs, was determined using Cufflinks. Comparisons between the groups were performed using the false discovery rate.

**Results:**

Fifty to 131 million high quality reads per specimen were obtained. Comparison of the reads to the NRVDB suggested that the demyelination and OND specimens had higher VHRs against some retroviral sequences compared with the controls. This was confirmed by retroviral domain averaging. Gene expression analysis showed differential expression among some HERV sequences. Single read mapping revealed one envelope and one reverse transcriptase sequence record that were significantly enriched among the demyelination samples compared to the normal controls. Less restrictive (comprehensive) read mapping showed that 2 integrase, 2 core, 2 envelope, and 3 KRAB sequences that were overexpressed in the demyelination group.

**Conclusions:**

These data demonstrate that some endogenous retroviral sequences are significantly overexpressed in these demyelination brain tissue specimens, but the magnitude of this overexpression is small. This is consistent with the concept of HERV activation as a part of the innate immune response.

## Introduction

Multiple sclerosis (MS) is a chronic demyelinating disease of unknown cause, which affects the brain and spinal cord of about 400,000 individuals in the U.S. A number of viral infections of the CNS can lead to demyelination, including distemper (dogs), measles (subacute sclerosing panencephalitis, SSPE, humans), and influenza (humans). [1]. Viruses have long been suspected as causative agents of MS, based on the epidemiology of the disease including geographic patterns, isolated outbreaks, and migration studies [2–5]. Novel viruses that cause human disease continue to be discovered including hepatitis C (1989), *corona virus* NL63 (2004), *bocavirus* (2005), and *rhinovirus* C group (2007) [6–9]. Novel human polyoma and arena viruses were recently identified as causes of serious human diseases by deep sequencing [10–12]. The National Multiple Sclerosis Society itself provides an excellent rationale for the search for viruses in MS and related diseases [13].

Past serology studies provided some evidence for the involvement of retroviruses in the pathogenesis of MS [14,15]. Other groups have identified human endogenous retroviruses (HERVs), including HERV-Fc1 and HERV-W, as possible MS pathogens [16–24]. Polymorphisms of human genes involved in the control of retroviral replication, including TRIM5, TRIM22, and BST2, are associated with higher risk of developing MS [25] and recent epidemiologic studies suggest that MS and HIV are mutually restrictive; that is, HIV patients develop MS less frequently than expected, adjusted for age, sex, and other factors [26–28].

Our group has used deep sequencing (also called next generation sequencing) to detect microbial sequences in donor brain tissues. This powerful new technique allowed identification of GBV-C in the brain of one MS subject and HSV or measles virus in the brains of several persons with encephalitis [29,30]. Deep sequencing is applied here to cryopreserved progressive MS and NMO brain specimens in comparison to normal brain and encephalitis brain specimen controls.

## Methods

Primary progressive MS cases were requested from the Human Brain and Spinal Fluid Resource Center (Los Angeles Veterans Administration, California) and the Rocky Mountain MS Center (Englewood, Colorado) brain repositories. Cases were selected by the directors of these two institutions. Specimens were studied from 11 persons with primary progressive MS (PPMS), one with secondary progressive MS (SPMS), and 2 persons with severe progressive neuromyelitis optica (NMO). Progressive MS and NMO cases were specifically selected because these tend to be the most severe subtypes of the MS-related diseases. Fourteen normal control and 5 frozen encephalitis brain specimens these resources were also studied. Two additional de-identified frozen encephalitis specimens were obtained from Dr. Don Gilden at the University of Colorado, Denver. The specimens were collected post-mortem within one day of death, either fresh frozen or snap frozen in liquid nitrogen, and were associated with a neuropathologic diagnosis. The research plan was submitted to the University of Utah Health Sciences IRB for review. This research was reviewed and approved by the University of Utah Health Sciences IRB, #00028658. Since this research involved only de-identified post-mortem material, it was found to be exempt from consent, review, and oversight.

The samples were assigned to one of three groups:

1. Demyelination (N=14)
  - Primary progressive MS (N = 11)
  - Secondary progressive MS (N = 1)
  - Neuromyelitis optica (N = 2)
2. Normal controls (n=14)
3. Other Neurologic Disease (OND) (n=7)
  - Herpes encephalitis (N = 3)
  - Other/unknown encephalitis (N = 2)
  - Subacute sclerosing pan encephalitis (N = 2)

The frozen brain specimens were handled as previously described [29] RNA was extracted from frozen brain with the RNeasy Lipid Tissue Mini Kit (Qiagen, Valencia, CA, Cat No./ID: 74804). The extracted RNA was DNase treated by incubating 15 minutes on the column. The resulting RNA was submitted for sequencing at the University of Utah High Throughput Genomics Shared Resource. Prior to sequencing, RNA was analyzed on an Agilent Bioanalyzer Nano chip (Agilent Technologies, USA) and evaluated for RNA abundance and integrity as previously described [29] Samples were reverse transcribed and libraries were prepared with the Illumina TruSeq kit (Illumina, San Diego, CA). To ensure the inclusion of possible viral RNA genomes, oligo dT selection was not performed. To avoid possible enrichment intrinsic biases, rRNA depletion was not performed.

The samples were sequenced and the sequencing reads were processed and aligned as previously described [29] Briefly, the reads were Illumina HiSeq 2500, 50 bp single-end. FASTQ format sequences were quality-filtered and high quality (HQ) sequences were retained. Reads that aligned to either the human genome (NCBI build GRCh37.p13) or human transcriptome, were removed from the HQ reads, yielding “screened reads” [31] Screened reads were aligned to the non-redundant viral database (NRVDB) using MegaBLAST (v2.2.26) with a word size of 28 bp [32] Viral hit counts (reads aligned to sequences in NRVDB) were normalized (divided) by the number of (HQ) high quality reads in a specimen.

The retroviral gene catalog (RVGC) was prepared by identifying regions of the human genome (build GRCh37.p13) with detectable similarity to core retroviral domains in the Gypsy 2.0 database (GyDB) using BLASTX [33,34] Alternative retroviral databases were considered for this analysis, and Gypsy was chosen due to the presence of annotations that allowed categorization of the sequences into retroviral domains. The BLASTx subprogram of version 2.2.26 of NCBI blast all was used with default word size and scoring and an expect cutoff of 0.1. Human genomic alignments with length 50% or longer than the subject retroviral domain were placed in RVGC. RVCG entries were named with arbitrary unique codes of the form: <domain type>_U<sequential_integer> (see S1 RVGC Table). For record keeping, the GyDB domain that was aligned to human sequence derived RVGC entry (recognition domain) and the source GI, starting position and length are also encoded in each RVGC fast a record name. Specifics of the compiled RVGC are available in Supplementary Data. Expression normalization was determined using the expression analysis pipeline Bowtie-Tophat-Cufflinks using only annotated splice sites [35]. The cufflinks norm mass parameter was used as the number of fragments for all FPKM metrics.

Statistical analysis of the demographic characteristics of the study population was performed using Vassar Stats [36], an open-source web application, and Microsoft Excel for Mac 2011. Differences among the groups were screened for significance by one way un-weighted ANOVA testing for continuous variables (age, year of collection, and post-mortem interval), and the two-tailed Chi-squared test with Yates correction for discrete variables (sex). For each taxon, viral hit rates (VHR) were compared between each demyelination and OND specimen and the set of controls using the Z-test with Bonferroni correction for multiple comparisons. Taxa where none of the demyelination or OND specimens VHRs were significantly different from controls were excluded from further analysis. Specific differences in VHRs between the groups were tested using the Mann-Whitney U-test or Tukeys HSD test. Correction for multiple comparisons was accomplished using the Benjamini–Hochberg procedure with a false discovery rate (FDR or q) of 0.05 [37]. The distribution of the sample types in the Retroviral Domain Discrimination Analysis was tested using the two-tailed Fishers Exact test for 2 × 2 tables [36].

## Results

### Subject Demographics

Characteristics of the study samples are shown in table 1. Fourteen demyelination samples were compared to 14 normal controls and 7 OND specimens. All patients in the demyelination group had severe and progressive clinical disease. Eleven were categorized as PPMS, two as NMO, and one as secondary progressive MS. The postmortem interval (PMI), time between death and collection of the brain sample, range was 2–26 hours. There were no significant differences in PMI between the groups (p=0.66). Age and sex information was available for all 14 controls, 13 of 14 demyelination cases, and 5 of 7 OND cases. The proportion of known females in the demyelination group 9/13 (69%) was not significantly different than the control group 6/14 (43%) (p=0.32). Ages of the subjects ranged from 37–93 years. The demyelination group was significantly younger (mean age 58 ± 13 years) than the normal controls (mean 71 ± 12 years, p=0.017). The specimens were collected between 1981 and 2010. The year of collection was not significantly different between the groups (p=0.29).

### Sequencing and Analysis

Deep sequencing yielded between 49.4 and 130.1 million 50 bp high quality (HQ) sequences per sample. The mean yield of HQ sequences was not different between the normal control (μ = 96.5 ± 7.1M) and demyelination (μ = 87.2 ± 16.6M) groups. The OND group yielded significantly fewer HQ sequences per sample (μ = 68.3 ± 17.0M) than both the normal control and the demyelination groups (p<0.01 for both comparisons).

**Figure 1:**
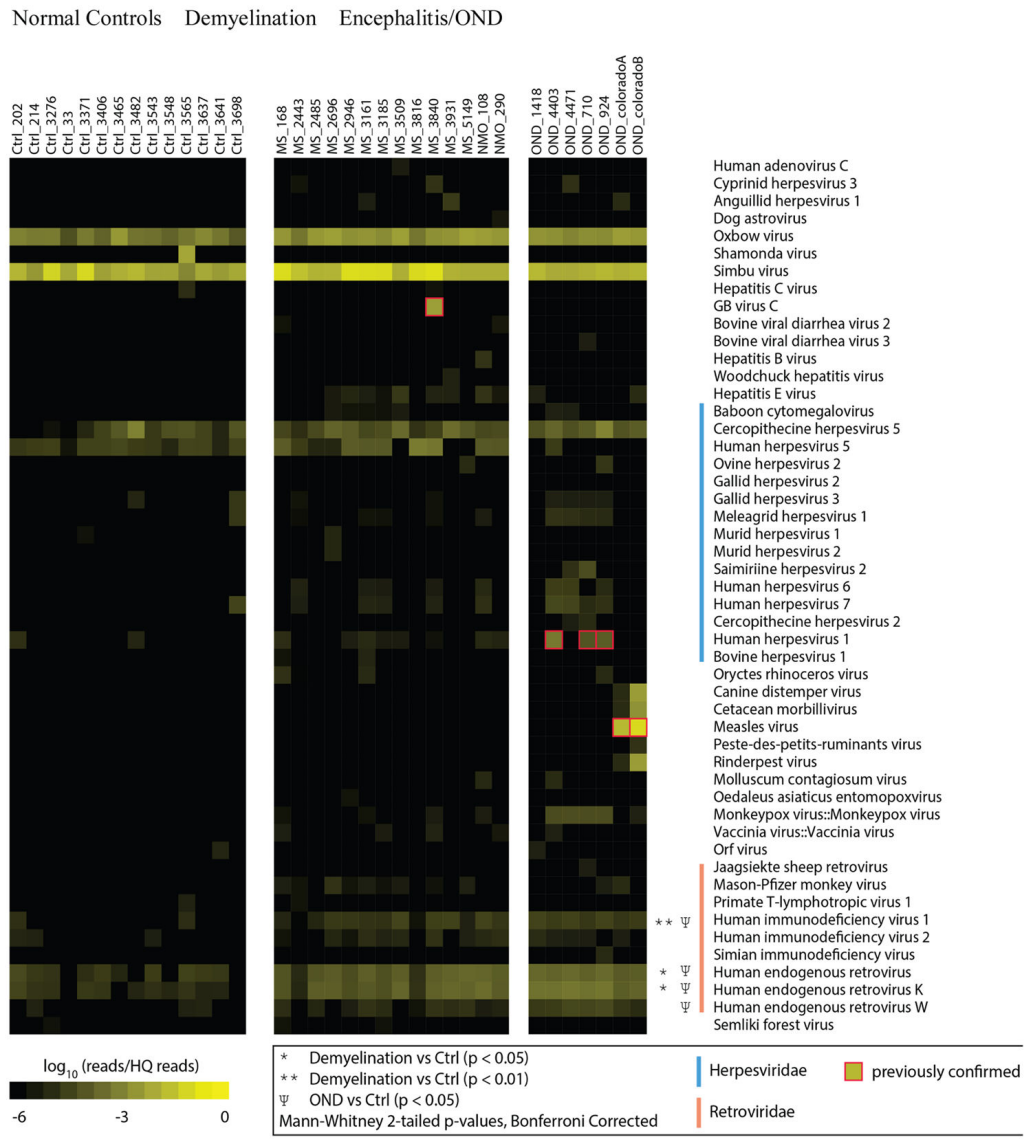
Hit Rate Comparison of Frozen Brain Specimens. RNA was extracted from 14 normal control, 14 demyelination (including 2 NMO), and 7 other neurologic disease (OND) cryopreserved brain specimens. cDNA libraries were prepared and deep sequencing was performed. High quality sequences were matched to the non-redundant viral database using Mega BLAST with a word size of 28 bp. Hits (matches to the database) were normalized for the total number of high quality reads in each specimen and log_10_ transformed, providing a ‘Log Hit Rate.’ Sequences mapping to non-vertebrate viruses (phages, plant and insect viruses) and cloning vectors were removed. Viral taxa not significantly enriched in any of the samples (e.g. VZV, EBV, and *Enteroviruses*) are not shown.

A heat map showing log-transformed HRs was generated from the sequencing analysis (Figure 1) [38]. Only viral taxa where one or more specimens are significantly overrepresented are displayed in the figure. Fifty viral taxa were over represented in at least one of the demyelination or OND specimens compared to the set of normal controls. In this manner, false positive alignments to human and cloning sequence were removed. *GB Virus C*, previously shown to be present only in specimen 3840, was the only non-retrovirus definitively present in any of the demyelination samples [30]. Bona fide and spurious viral alignments within the OND samples have been previously described [29].

Among the 50 viral taxa overrepresented in at least one OND or demyelination samples, 17 were herpes viruses and 9 were retroviruses. Statistical comparisons (corrected for multiple comparisons) of VHRs between all members of the groups were performed. This analysis revealed that only 3 retroviral taxa that were significantly over expressed in the demyelination group compared to the control group: human immunodeficiency virus 1 (HIV-1), human endogenous retroviruses (family), and human endogenous retrovirus K. There were no AIDS or HIV patients in the cohort; thus the high VHR to HIV-1 was inferred to be caused by the 50 bp reads aligning to sequences similar to HIV-1. None of the 9 herpes virus candidate taxa were significantly different between the groups.

### Retroviral Analysis

The apparent retroviral sequence enrichment in the demyelination group led to a more inclusive retroviral sequence analysis. A new database called the retroviral gene catalog (RVGC) was prepared (see Methods). RVGC contains endogenous sequences similar to protein-coding retroviral genes: GAG, RT, ENV, etc. Specific information about all the sequences records in the RVGC, including GenBank identifiers, length, and location is available (S1 RVGC Table).

Reads aligning to the RVGC database were binned according to retroviral domains type (e.g. GAG, RT, ENV). GAG refers to the viral core, RT to the reverse transcriptase, and ENV the envelope. The functions of the SCAN domain are not well understood and KRAB is probably a transcriptional repressor [39]. CHR refers to the chromo domain found at the C-terminal end of many retro transposon integrases [40].

**Figure 2:**
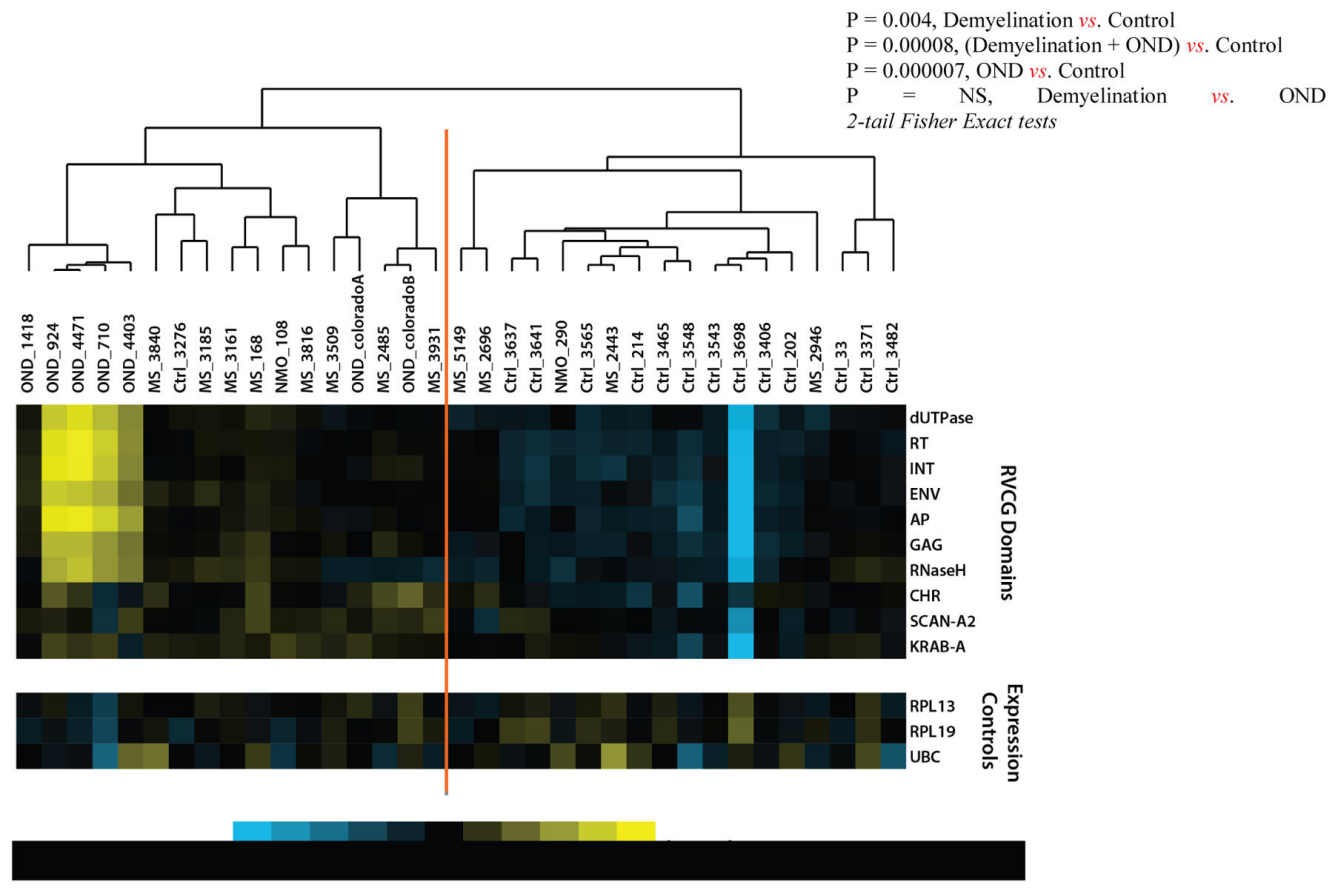
Retroviral Domain Discrimination Analysis. Sequencing hits (matches) to the RVGC database were binned into retroviral domains. Mean log_2_ transformed and centered hit rates to the retroviral domain types in RVGC for the demyelination (MS), OND and normal control specimens. The results are clustered using Cluster 3.0 [41], showing distinctly different expression patterns in most demyelination, control and OND specimens.

Mean domain-type hit rates (HRs) for the demyelination, NMO and control samples were log_2_ transformed and centered in the domain-type axis (Figure 2). These values were hierarchically clustered (Pearson correlation) and compared between the demyelination and normal control specimens [41]. The resulting sample clustering reveals the relative separation of demyelination and control specimens into the dominant nodes of the cluster (e.g. 9/10 demyelination samples cluster left, 12/16 control samples cluster right; p=0.004). This data set shows evidence of broad retroviral gene over expression in the brains of the demyelination subjects compared with normal controls. However, the magnitude of the retroviral over expression was small, less than 2-fold, and was not evident for any single RVGC sequence. This retroviral gene expression pattern was more pronounced among the OND (encephalitis) samples than in the demyelination group. Expression of the neural tissue control genes RPL13, RPL19, and UBC was not significantly different between the 3 groups.

Additional analysis showed that some specific retroviral genes are significantly over expressed in the demyelination (N=14) and PPMS only (N=11) groups compared with the normal controls (n=14). Two mapping procedures were employed: “Best Alignment” where each read was mapped to the RVGC only once to its best match, and “Comprehensive Alignment” where every reported Bowtie 2 alignment was counted. The results of this analysis are displayed in Table 2. Only one envelope gene and one RT gene were significantly over expressed by the best alignment procedure. Several integrase, GAG, and envelope genes, along with 3 KRAB genes, were over expressed by the comprehensive alignment procedure; limiting the analysis to the 11 PPMS specimens showed those 2 ENVs and 3 GAGs were over expressed compared to controls. The HERV annotations for these over expressed genes are shown in Table 2.

## Discussion and Conclusion

This study employed deep sequencing and metagenomic analysis techniques to comprehensively investigate retroviral expression among frozen demyelination brain samples compared with normal controls and OND (encephalitis) controls. The results show some over expression of HERVs in general among most domains (Figure 2). Over expressed HERV and KRAB sequences were specifically identified corresponding to several retroviral domains, including core, envelope, integrase, and reverse transcriptase (Table 2). These results support the hypothesis that retroviral sequences are over expressed in demyelinated brain samples compared with normal brain. However, the magnitude of the observed retroviral domain over expression was small, less than 3-fold, and the pathological significance of this observation is unknown.

The data from this study are consistent with the concept that HERV over expression is part of the human immune response. Other groups have identified the MSRV (HERV-W) or HERV-Fc1 as possibly contributing to the pathogenesis of MS [16,17,19,20,24,42]. Interestingly, the present sequencing study did not specifically confirm these findings (Figure 1). Instead, some other HERV genes from a variety of sources were shown to be significantly over expressed (Table 2). This highlights the difficulties inherent with these studies where multiple similar HERVs have been incorporated into the human genome. The gene expression mapping performed here relies on annotated sequences from the human genome build 37. The data generated in the present sequencing study is comprehensive across the entire human genome, but it is likely to be less specific and less quantitative than qPCR.

Human endogenous retroviruses (HERVs) are remnants of ancient retroviral infections of the host germ line that are transmitted vertically from parents to their offspring. The utility of these elements within the human genome remains largely speculative, although at least one HERV codes for syncitin, an important protein that allows for development of the placenta [43].

Interestingly, some animal ERVs efficiently interfere with the replication of related exogenous retroviruses [44,45]. Sheep have been used to study the evolution of ERVs within a mammalian host due to the presence of related exogenous and endogenous retroviruses. The exogenous (i.e., horizontally transmitted) oncogenic *retrovirus*, *Jaagsiekte sheep retrovirus* (JSRV), causes fatal lung cancers. A closely related provirus, JSRV-20, entered the host genome within the last 3 million years during speciation within the genus *Ovis. Endogenous JSRV* has a defective Gag polyprotein resulting in a transdominant phenotype that blocks the replication of the closely related *exogenous JSRV* [46–48]. That is, the expression of an endogenous retrovirus effectively blocks a fatal infection with an exogenous retrovirus. This animal data strongly suggests that endogenization and selection of ERVs is a mechanism used by the host to fight retroviral infections. Support for this concept in humans is displayed by the increased expression of (endogenous) HERV-K in patients infected with the (exogenous) retrovirus HIV, where HERV-K envelope is neuroprotective [49,50].

Most of OND specimens were from patients with either *herpes* (3) or *measles* virus (2) encephalitis. The HSV infected specimens (4403, 710, 924) displayed the highest levels of HERV domain expression, as shown in Figure 2. This is consistent with the results of other groups that have shown HSV stimulates reverse transcriptase in PBMCs from MS patients [51], and HERV-W expression is induced by HSV1 in cell cultures [52,53].

One limitation of this deep sequencing method is that the RNA extractions are not completely DNA free, despite a DNAse treatment step. Qubit analysis of extracted brain specimens revealed that 1–5% of the analyzed material is DNA retained from the original sample. Prior to sequencing, the RNA were also analyzed on an Agilent Bioanalyzer Nanochip (Agilent Technologies, USA) and evaluated for RNA size, abundance and integrity. This provided relatively high quality RNA for the subsequent analysis, but it cannot be determined with absolute certainty that retained DNA did not affect the results of the study. Another limitation of the study is the post-mortem interval necessarily associated with these samples obtained from deceased human donors. While there was no detectable difference in the PMI between the demyelination and control specimens, this interval likely allowed some RNA to degrade in all the samples. This likely did interfere with the detection of HERV and other retroviral sequences during the sequencing reactions.

## Supplementary Material

Table S1

## Figures and Tables

**Table 1:** Characteristics of the sequenced frozen brain specimens.

Group	Specimen	PMI	Age	Sex	Collection Year	Clinical History	Specimen Location	Neuropathology Reading	HQ Reads
**Normal Control**	202	4	57	M	1983	Post-surgical infection	cerebrum	normal	91.5
	214	4	56	M	1981	Heart Disease	cerebrum	normal	94.7
	3276	19	54	M	2002	Heart Disease	frontal WM	normal	93.9
	33	5	69	M	1983	Lung Disease	cerebrum	normal	89.1
	3371	16	52	M	2002	Lung Cancer	frontal WM	normal	96.8
	3406	20	72	F	2002	Heart Failure	temporal WM	normal	89.3
	3465	20	93	F	2002	Bleeding	temporal WM	normal	105.2
	3482	14	79	F	2003	Heart Disease	temporal WM	normal	97.5
	3543	12	73	F	2003	Lung Disease	parietal WM	normal	105.2
	3348	9	76	F	2002	Heart Disease, Diabetes	frontal WM	normal	102.7
	3465	11	76	M	2003	Heart Failure	occipital WM	normal	107.3
	3637	13	76	M	2003	Pneumonia	frontal WM	normal	102.5
	3641	20	76	M	2003	Stomach and Liver Cancer	parietal WM	normal	85.3
	3698	17	84	F	2003	Breast Cancer	temporal WM	normal	90.3
**Demyelination (Progressive MS and NMO)**	168	3	37	F	1984	PPMS	cerebrum	reactivated MS	89.7
	2443	26	49	F	1997	PPMS	periventricular WM	lymphocytic cuffing	99
	2485	9	69	M	1997	PPMS	frontal WM	active MS	63.3
	2696	21	86	F	1998	PPMS	periventricular WM	chronic MS plaque	130.9
	2946	15	59	M	1999	PPMS	periventricular WM	chronic perivascular inflammation	88.9
	3161	20	51	F	2001	Secondary Progressive MS	periventricular WM	active MS with lymphocytic cuffing	89.9
	3185	14	50	M	2001	PPMS	periventricular WM	chronic MSplaque	91.1
	3509	11	74	F	2003	PPMS	periventricular WM	chronic MS plaque	87.2
	3816	21	47	F	2004	PPMS	frontal WM	macrophages and lymphocytic cuffing	91
	3840	23	61	F	2003	PPMS	frontal WM	macrophages and lymphocytic cuffing	95
	3931	10	74	F	2004	PPMS	periventricular WM	chronic active disease	73.8
	5149	8	48	M	2010	PPMS	frontal WM	active MS	66.4
	108	2	56	F	1998	NMO	midbrain and pons	demyelination with necrosis	72.5
	290	NA	NA	NA	NA	NMO	corpus callosum	demyelination consistent with NMO	82.3
**OND Control**	1418	5	68	M	1988	MS-like illness†	Frontal cortex	chronic encephalitis	76.8
	4403*	18	77	F	2006	HSV encephalitis, stroke	Frontal cortex	active encephalitis	99.5
	4471	12	73	F	2007	Rasmussen’s encephalitis	Frontal cortex	gliosis without active inflammation	49.4
	710*	8	58	M	1983	HSV encephalitis, chronic lymphocytic leukemia	Frontal cortex	active encephalitis	50.1
	924*	24	86	M	1985	HSV encephalitis	Frontal cortex	chronic encephalitis	67.9
	coloradoA	NA	NA	NA	NA	SSPE (measles)	NA	NA	67.9
	coloradoB	NA	NA	NA	NA	SSPE (measles)	NA	NA	66.9

NA = information not available

NMO = neuromyelitis optica

OND = other neurologic disease

PMI = post-mortem interval (hours)

HQ reads = high quality reads (in millions)

PPMS = primary progressive multiple sclerosis

SSPE = subacute sclerosing panencephalitis (measles infection)

WM = white matter

* previously diagnosed as HSV encephalitis

† MS not confirmed by pathology

**Table 2:** Specific Retroviral Genes Overexpressed in the Demyelination Group Fifty bp reads were aligned to all annotated HERV sequence in NCBI/GenBank using the Bowtie2application with the default settings for end to end alignment. Mean expression levels (reads per million per kilobase, FPKM) are shown for the demyelination (progressive MS and NMO) groups. Reads were mapped either: 1) singly, to their best matching RVCG sequence (Best Alignment) or, 2) using every reported Bowtie2 alignment to the RVGC (Comprehensive Alignment). P-values and False Discovery Rates (FDR) are reported, along with the ratio of the mean hit rates between the experimental and control groups. Retroviral associations are per the annotation in the Gypsy 2.0 database.

Method	Group	Gene	Expression 1	Ratio2	P-value	FDR (q) D:C3	Recognition Domain
**Best Alignment**	Demyelination	ENV-U3	958	1.7	0.0001	0.006	K-HERV
	(N=14)	RT-U105	300	1.7	0.0001	0.032	MMTV
	PPMS only	ENV-U3	952	1.7	0.0002	0.017	K-HERV
	(N=11)	RT-U105	311	1.8	0.00005	0.014	MMTV
**Comprehensive Alignment**	Demyelination	INT-U176	775	1.7	0.0001	0.036	MuERV-L
	(N=14)	INT-U45	94	2.8	0.0003	0.044	
		GAG-U21	52	2.5	0.0003	0.009	K-HERV
		GAG-U22	43	2.3	0.0011	0.018	HERV-K10
		ENV-U59	1540	1.9	0.0009	0.037	RTVL-Ia
		ENV-U3	960	1.7	0.00007	0.006	K-HERV
		KRAB-U15	9880	1.8	0.001	0.017	KRAB
		KRAB-U13	1027	1.7	0.001	0.017	
		KRAB-U9	1982	1.5	0.006	0.037	
	PPMS only	ENV-U3	952	1.7	0.0002	0.019	K-HERV
	(N=11)	ENV-U59	1617	2	0.0009	0.04	RTVL-Ia
		GAG-U21	56	2.7	0.00009	0.003	K-HERV
		GAG-U22	47	2.5	0.0006	0.009	HERV-K10
		GAG-U8	242	1.5	0.004	0.041	HERV-E

1 Expression is described as FPKM (Fragments Per Kilobase of transcript per Million mapped reads) × 1000

2 Ratio = mean expression in the Demyelination or PPMS group divided by mean expression in the Control group

3 False Discovery Rate (FDR) q-values were calculated over each domain type separately (e.g. RT, ENV, GAG, etc.)
